# Noncommunicable Diseases and Nutrition Disorders in Occupational Health

**DOI:** 10.3390/nu18030442

**Published:** 2026-01-29

**Authors:** Blanca Herrero-Salas, Javier Sanz-Valero

**Affiliations:** National School of Occupational Medicine, Carlos III Health Institute, 28029 Madrid, Spain; mblanca.herrero@isciii.es

## 1. Introduction

Bernardino Ramazzini, considered the father of occupational medicine, was a pioneer in formulating principles aimed at protecting workers’ health [1]. His contributions, which were notably ahead of their time, included recommendations to reduce working hours and establish rest periods and correct body posture, directly anticipating the fundamentals of modern ergonomics, as well as promoting proper nutrition and recognizing poverty as a determining factor in the development of various diseases [2]. These proposals, framed in 17th-century Europe, anticipated contemporary debates on the close relationship between working conditions and health.

Ramazzini’s vision of placing health at the heart of work paved the way for our understanding of how physical, social, and economic factors affect workers’ well-being. This historical perspective enables us to relate his ideas to contemporary issues, where chronic diseases and nutritional issues have become critical factors in an individual’s ability to perform a job.

Thus, noncommunicable diseases (NCD) and nutrition disorders (ND) have become key determinants of the health of the working population, increasing the burden of disability, absenteeism, and presenteeism. Evidence links both malnutrition by default (malnutrition, micronutrient deficiencies) and by excess (overweight, obesity) with poorer control of cardiovascular, metabolic, and musculoskeletal diseases, which affect functional capacity and work performance. In this context, occupational health can no longer be limited to the control of classic risks but must explicitly integrate the prevention and management of nutritional problems and their associated comorbidities [3].

The scale of the problem is global. It is estimated that one in three people worldwide suffers from at least one form of malnutrition, which directly affects morbidity, mortality and labor productivity. In low- and middle-income countries, malnutrition and iron deficiency anemia persist among workers. In high-income countries, meanwhile, obesity and other chronic diseases related to unhealthy diets and sedentary lifestyles are more prevalent. These nutritional inequalities result in considerable economic losses due to decreased productivity, increased workplace accidents and greater use of occupational health services [4].

Furthermore, ND includes not only nutritional excess or deficiency but also eating behavior disorders, which have traditionally been underestimated in the workplace. Mitchison et al. [5] indicated that these disorders significantly affect mental health, sleep quality, and concentration, factors directly related to productivity and safety at work. Likewise, work stress, high psychological demands, and adverse organizational environments can act as triggers or perpetuators of dysfunctional eating behaviors, establishing a bidirectional relationship between work and nutritional disorders [6].

Despite the relevance of the issue, nutrition continues to be an insufficiently incorporated component of occupational safety and health policies and regulations. Although most workers consume at least one main meal at their workplace, the integration of nutrition into occupational health regulatory frameworks remains limited, despite its proven impact on health and work performance [7]. This regulatory gap underscores the urgency of promoting a more comprehensive approach that considers nutrition as a key determinant of occupational health.

## 2. Perspectives and Scientific Gaps in the Relationship Between Nutritional Health and the Work Environment

The relationship between nutritional status and occupational health has gained relevance, but significant fragmentation persists in the scientific literature. Despite the impact of NCD and ND on workers’ health and productivity, there is a shortage of longitudinal studies evaluating how specific working conditions (shift work, emotional or professional exhaustion, heat stress, or extreme sedentary lifestyles) interact with these pathologies in the long term. Most current research is limited to cross-sectional designs that do not allow for establishing a clear causality between the work environment and the development of complex metabolic disorders [8,9].

A critical gap has been identified in the personalization of nutritional interventions in the workplace. Although workplace health promotion programs are common, evidence of their effectiveness in populations with specific nutritional needs due to preexisting conditions is limited. The literature suggests that the lack of standardized protocols and the heterogeneity of jobs make it difficult to implement evidence-based dietary strategies that consider both the physical workload of the employee and their individual clinical profile [10,11].

Conversely, contemporary research tends to treat nutrition and occupational toxicology as distinct disciplines, limiting our understanding of their interaction within the body. This separation hinders the recognition that exposure to toxins in the workplace can modify key metabolic pathways, alter the bioavailability of nutrients or increase specific dietary requirements [12]. Similarly, a person’s nutritional status can affect the absorption, distribution, and elimination of toxic substances, thereby influencing their vulnerability or resistance. Integrating these two perspectives would enable the development of more effective preventive strategies and provide a more comprehensive view of occupational health [13,14]. This integration is not only methodologically necessary but also essential for developing more accurate preventive strategies and achieving a more comprehensive understanding of occupational health in the current context.

It is also important to acknowledge the lack of research examining the impact of job and food insecurity on workers’ health. Most scientific research focuses on corporate environments in developed countries, failing to analyze how nutritional disorders caused by deficiencies or excesses affect workers in the informal sector or in emerging economies [15]. To understand how wage structure and access to quality food act as fundamental social determinants of contemporary occupational health, it is imperative to integrate robust socioeconomic metrics [16,17].

There is also a lack of documentation regarding the impact of teleworking and the digitization of working hours on eating behavior. The transition to hybrid working models has blurred the boundaries between domestic and professional spaces, thereby altering circadian eating rhythms and access to controlled food environments [18]. It is urgent that we investigate how “permanent availability” influenced by technology affects emotional hunger and diet quality, and the role that food delivery platforms play in the metabolic health of remote workers [19].

Few studies integrate the microbiota–gut–brain axis into occupational well-being. Although work-related stress and professional burnout are known risk factors for gastrointestinal disorders, research has not yet sufficiently explored how modulation of microbiota through diet can act as a protective factor against burnout or cognitive impairment. Incorporating digestive health as a pillar of intellectual performance and emotional resilience would represent a paradigm shift in contemporary occupational medicine [20,21].

In addition to the above, there are some additional gaps that could be addressed in the context of occupational health research from a public health and occupational medicine perspective. First, the intersection between workloads and caregiving tasks—the so-called “double shift”—affects the quality of workers’ diets and their time availability, a phenomenon that has scarcely been analyzed from an occupational health perspective [22]. Second, analysis of the workplace environment (its physical design), including the availability of vending machines and corporate cafeterias, often ignores the way in which the layout of food options—i.e., the decision architecture—influences worker behavior beyond their individual will [23,24].

## 3. Comprehensive Interventions in the Workplace

In 2008, the World Health Organization recommended implementing active health promotion policies, including workplace nutrition programs [25]. Based on these recommendations and the latest scientific literature, we propose interventions to help bridge the gaps in scientific research on the relationship between nutritional health and the work environment.

The relationship between nutritional status and occupational health has become increasingly important in recent years, especially in a context marked by the rise in NCD, intensified work demands, and the digital transformation of work. However, scientific literature continues to show significant fragmentation. Although NCD and occupational diseases directly affect workers’ productivity, performance, and overall health, there remains a notable shortage of longitudinal studies analyzing how specific working conditions, such as shift work, emotional exhaustion, heat stress, or extreme sedentary lifestyles, interact with these pathologies over time. For this reason, authors such as Kivimäki et al. [26], Lavigne-Robichaud et al. [27], and Prakash et al. [28], among others, have emphasized that the predominance of cross-sectional designs limits the ability to establish solid causal relationships between the work environment and the development of certain pathologies.

To address this research gap on the relationship between nutritional status and occupational health, it is imperative to move toward a more robust methodological approach that prioritizes the design of prospective cohort studies that allow for the monitoring of changes in nutritional status in relation to occupational exposure variables over multi-year cycles.

Collaboration between industry and academia could guarantee access to stable population samples, enabling science to not only describe occupational health issues but also to propose predictive models for them. Creating collaborative networks between nutrition specialists and risk prevention departments would allow for clinical practice guidelines to be validated, transforming workplaces into controlled ecosystems for obtaining robust evidence on the effectiveness of therapeutic diets for working populations. Promoting continuous training in personalized nutrition for occupational health professionals could improve the quality and impact of these interventions. Using wearable technologies and continuous monitoring systems, such as activity sensors, digital dietary recording devices, and circadian analysis tools, would allow for high-resolution longitudinal data to be collected and improve our understanding of the physiological mechanisms linking the work environment to metabolic risk [29,30].

Another critical gap identified in the contemporary literature is the lack of personalization of nutritional interventions in the workplace. Although workplace health promotion programs have expanded over the past decade, their effectiveness in populations with specific nutritional needs—such as workers with obesity, type 2 diabetes, or cardiovascular disease—remains limited. Recent studies indicate that the absence of standardized protocols and the heterogeneity of jobs make it difficult to implement evidence-based dietary strategies that consider both the physical workload of the employee and their individual clinical profile [31,32]. The integration of personalized nutrition approaches, supported by genetic, metabolomic, and behavioral analyses, could improve adherence and clinical outcomes in the workplace.

Furthermore, current research continues to address nutrition and occupational toxicology as separate fields, limiting understanding of their biological interactions. However, recent research has shown that exposure to environmental and occupational toxins—such as heavy metals, volatile organic compounds, or pesticides—can alter key metabolic pathways, modify the bioavailability of nutrients, and increase specific dietary requirements [33]. In turn, nutritional status can modulate the absorption, distribution, and elimination of toxic substances, influencing worker vulnerability. The integration of toxic–nutritional models therefore represents a priority line of research for the development of more effective preventive strategies.

Another area that has not been sufficiently explored is the impact of job and food insecurity on workers’ health. Much of the existing scientific research continues to focus on corporate environments in high-income countries, overlooking workers in the informal sector or emerging economies. However, recent studies have revealed a link between food insecurity and an elevated risk of obesity, diabetes, chronic stress, and deteriorating mental health, establishing this phenomenon as a critical social determinant of contemporary occupational health [34]. To address this gap, robust socioeconomic metrics and intersectional approaches must be incorporated to enable analysis of the impact of wage structure, job stability and access to quality food on workers’ nutritional status.

The digitization of work and the growth in remote working constitute an emerging environment that requires urgent scientific attention. The shift towards hybrid working models has blurred the boundaries between domestic and professional spaces, thereby altering circadian eating rhythms, availability of controlled eating environments and emotional relationships with food. Studies by Sato et al. [35] and Hoshi et al. [36] have shown that teleworking can lead to irregular eating patterns and increased consumption of ultra-processed foods, particularly in situations involving high technological demands. Additionally, the growth of food delivery platforms raises questions about their potential impact on the dietary quality and metabolic health of remote workers.

Similarly, the absence of studies that integrate the microbiota–gut–brain axis into the analysis of occupational well-being represents a valuable research opportunity. Recent evidence has shown that gut microbiota plays a key role in regulating stress, systemic inflammation, and cognitive function, suggesting that its modulation through dietary interventions could act as a protective factor against burnout and cognitive impairment associated with chronic work-related stress [20,37]. Incorporating this perspective into occupational medicine could transform our understanding of intellectual performance and emotional resilience in demanding work environments.

Finally, from a public health perspective, it is also necessary to address additional gaps such as the intersection between workloads and caregiving tasks, the so-called “double shift,” which affects dietary quality and the availability of time for food preparation, especially among working women. Likewise, analysis of the food environment in workplaces, including the offerings in vending machines and corporate cafeterias, often ignores how decision architecture influences eating behavior beyond individual willpower. Interventions based on nudging and redesigning the food environment have proven effective in improving the quality of food choices in work contexts [38,39].

Together, these lines of action would overcome the current fragmentation of the literature and move towards a multidisciplinary approach integrating biological, social and environmental dimensions. Consolidating a robust field of research in nutrition and occupational health requires collaboration between nutritionists, epidemiologists, toxicologists, occupational psychologists, prevention technicians, occupational medical doctors and nurses, engineers, and public health officials. It also requires the development of innovative methodologies that capture the complexity of the contemporary work environment.

## 4. Corollary

Historical and contemporary evidence confirms that ND and NCD are key determinants of occupational health, directly impacting functional capacity, productivity, and the sustainability of work systems. From Ramazzini’s pioneering vision to the current challenges of digitalization, job insecurity and socioeconomic inequalities, occupational health cannot remain limited to traditional risk control; it must incorporate the prevention, treatment and personalization of nutritional disorders on a structural level. However, the fragmentation of the literature, the scarcity of longitudinal studies, the weak integration between nutrition and toxicology, and the limited consideration of emerging factors—such as teleworking, food insecurity, microbiota, and double shifts—reveal significant scientific and regulatory gaps. Overcoming these gaps requires a multidisciplinary, methodologically robust, and socially sensitive approach that considers biological, psychological, environmental, and economic factors. Only through collaboration between academia, prevention systems, and public policy will it be possible to transform the workplace into a strategic space for promoting nutritional health, reducing NCD, and strengthening the well-being and resilience of the working population in the contemporary labor context.

## Data Availability

No new data was generated in the production of this Editorial.

## Abbreviations

The following abbreviations are used in this manuscript:

NCD	Noncommunicable diseases
ND	Nutritional disorders
